# Effect of exercise training on cardiac function and glucose metabolism in the ischemic border zone: insights from multi-modal imaging techniques

**DOI:** 10.3389/fcvm.2025.1583206

**Published:** 2025-05-30

**Authors:** Jun Zhang, Chunrong Jin, Xiao Han, Ping Wu, Jianbo Cao, Sheng He, Li Li, Ruonan Wang, Min Zhang, Yuxin Xiao, Hongju Guo, Tianshuo Zhang, Zhifang Wu, Sijin Li

**Affiliations:** ^1^ Department of Nuclear Medicine, First Hospital of Shanxi Medical University, Taiyuan, Shanxi, China; ^2^ Collaborative Innovation Center for Molecular Imaging of Precision Medicine, First Hospital of Shanxi Medical University, Taiyuan, Shanxi, China; ^3^ Department of Cardiology, First Hospital of Shanxi Medical University, Taiyuan, Shanxi, China; ^4^ Shanxi Key Laboratory of Molecular Imaging, Shanxi Medical University, Taiyuan, Shanxi, China; ^5^ Department of Radiology, First Hospital of Shanxi Medical University, Taiyuan, Shanxi, China; ^6^ Department of Biochemistry and Molecular Biology, School of Basic Medicine, Shanxi Medical University, Taiyuan, Shanxi, China; ^7^ School of Pharmacy, Shanxi Medical University, Taiyuan, Shanxi, China

**Keywords:** acute myocardial infarction, cardiac function, remodeling, myocardial glucose metabolism, RNA sequencing, exercise

## Abstract

**Aims:**

This study aimed to evaluate the impact of early exercise following acute myocardial infarction (AMI) on cardiac function, myocardial remodeling, glucose metabolism, and its molecular changes using cardiac magnetic resonance (CMR) imaging and positron emission tomography (PET).

**Methods and results:**

Thirteen rats (MI-exercise, MIE) underwent an 8-week treadmill exercise training initiated 1 week after AMI. Longitudinal assessments were conducted using 7T CMR and ^18^F-FDG PET/CT imaging at baseline, 4 weeks and 8 weeks following the commencement of exercise. Molecular and pathological analyses, including qPCR and Western blot, were conducted to evaluate mRNA and protein expression related to glucose metabolism. Exercise training led to significant improvements in stroke volume (SV), left ventricular ejection fraction (LVEF), and fraction wall thickening (WT%) from 4 weeks onward, as assessed by CMR, which strongly correlated with increased myocardial glucose uptake, as measured by ^18^F-FDG PET (*P* < 0.05). Histological analysis revealed a marked reduction in inflammatory cell infiltration and fibrosis percentage (MIE vs. MIC: 23.42 ± 5.4% vs. 40.63 ± 8.9%, *P* < 0.05), accompanied by an increase in myocardial cross-sectional area (MIE vs. MIC: 817.15 ± 36.54 μm^2^ vs. 379.28 ± 67.99 μm^2^, *P* = 0.002). RNA sequencing demonstrated upregulation of pathways associated with cellular metabolism. Additionally, the expression levels of GLUT4 and PFKFB3 mRNA and proteins were significantly elevated following exercise training.

**Conclusions:**

Early exercise post-AMI, as assessed by CMR and PET imaging, significantly improved cardiac function, reduced myocardial remodeling, and enhanced glucose metabolism. These benefits were mediated through the upregulation of GLUT4 and PFKFB3 expression, underscoring the potential of exercise as a therapeutic strategy in post-AMI management.

## Introduction

Acute myocardial infarction (AMI) is a leading cause of heart failure characterized by ischemia and hypoxia in cardiomyocytes, which disrupt energy metabolism and contribute significantly to premature death and disability worldwide (1, 2). One of the critical drivers of structural remodeling and functional impairment following AMI is the disruption of myocardial energy supply. Under normal physiological conditions, the energy required for cardiac contraction and the maintenance of ion channel stability predominantly derives from fatty acid (FA) β-oxidation, with glucose oxidation and glycolysis serving as secondary energy sources. However, ischemic and hypoxic conditions suppress fatty acid oxidation, shifting energy metabolism toward glucose metabolism, which generates ATP more efficiently under these circumstances (3–5). Currently available pharmacological treatments, such as β-blockers, ACE inhibitors/ARBs, and aldosterone antagonists, primarily aim to reduce myocardial energy consumption. Despite these advancements, the energy supply to the heart post-AMI remains inadequate. Additionally, emerging evidence suggests that exercise training may serve as a promising non-pharmacological intervention by modulating energy metabolism (6–8). However, the clinical adoption of exercise training post-AMI remains controversial due to ongoing debates regarding its safety and effectiveness, which have limited its integration into long-term management strategies for AMI patients.

To address this, precise and dynamic methods are required to evaluate cardiac function and myocardial metabolism, providing evidence to substantiate the benefits of early exercise post-AMI and inform clinical practice. Moreover, cardiac magnetic resonance (CMR), recognized for its high spatial resolution and absence of radiation, has emerged as the gold standard for assessing cardiac structure and function. It enables accurate, quantitative tracking of cardiac remodeling and hemodynamic changes (9, 10). Additionally, 2-deoxy-2-[^18^F] fluor-D-glucose (^18^F-FDG) PET imaging is widely used to evaluate myocardial glucose uptake, offering a non-invasive and repeatable approach to monitor glucose metabolism dynamics in myocardial cells.

In this study, we aimed to utilize CMR and PET imaging techniques to comprehensively and accurately assess the effects of exercise training on cardiac function, myocardial remodeling, and glucose metabolism in a rat model of AMI. Furthermore, we sought to explore the underlying biological changes induced by exercise training to support its clinical application as an effective post-AMI intervention.

## Methods

### Ethical approval and animals

This study was carried out in accordance with ARRIVE (Animals in Research: Reporting *in vivo* Experiments) guidelines and approved by the Animal Care Committee of the Shanxi Medical University (DWLL-2024-012).

### Experimental groups and model validation

All animals were maintained with artificial 12:12 h equivalent light–dark cycle environment with 22 ± 2℃ constant temperature, 50 ± 5% relative humidity and free access to food and water. Thirty-nine male Sprague–Dawley rats aged 7–8 weeks [SPF (Beijing) Biotechnology Co., Ltd.] were included in this study. The model of AMI was induced by left anterior descending coronary (LAD) artery ligation according to the previous experimental procedure (11). The Sham-operated rats (*n* = 13) were manipulated in the same procedure, with no actual ligation of LAD. One week after operation, rats with infarct sizes between 30% and 35% of the left ventricular free wall pathologically confirmed were used in the following experiments. The surviving rats after AMI were randomized into MIC group (MI-sedentary control, *n* = 13) or MIE group (MI-exercise, *n* = 13).

### Exercise training and exhaustion test

After 1 week of acclimatization to treadmill exercise (a speed of 10 m/min for 10 min per day), the formal exercise training began at 1 week after AMI and continued for 8 weeks (5 days of treadmill per week at a speed of 16 m/min for 50 min/day, including preheating for 5 min at 10 m/min). MIC or Sham rats were assigned to remain sedentary during the whole experiments. After 8 weeks of exercise training, an exhaustion test was performed on each group of rats as the previous method (12). Exhaustion was defined as the inability of a rat to resume running within 10 s after direct contact with the electric-stimulus grid or the number of electrical stimulation >40. The running time and the running distance were recorded.

### Assessment of cardiac functions and myocardial remodeling by CMR

CMR were acquired with a 7T magnet (Bruker 70/20USR, Germany) equipped with a cardiac specific surface coil (RF ARR 300 1H R.HRT.4*1 ROAD) and 8.6 cm inner diameter transmitting coil (RF res 300 1H 112/086 QSN TO AD/RF ARR 300 1H R.HRT.4 × 1 ROAD). Rats were anesthetized using 2% isoflurane with 2–3 l/min of oxygen. The isoflurane concentration was then adjusted to 1.25%–1.5% based on the respiratory rate. After anesthesia, the animals were positioned supine on the scanning bed. ECG electrodes were inserted subcutaneously into the limbs. Heart rate (HR), respiratory rate and body temperature were monitored (Small Animal Instruments Inc, SAII, U.S.A.) and recorded during image acquisition. To assess left ventricular (LV) anatomy and function as well as myocardial remodeling, cine CMR were acquired in the short-axis views. The CMR data were converted into polar maps and divided into segments using a standard 17-segment model to obtain the WT%. The sequence parameters were as follows: 2 ms of echo time, 48 ms of repetition time, 8 of repetitions, 18° of flip angle, 8 of frames, 60 mm × 60 mm field of view (FOV), 192 × 192 of matrix, 1.5 mm of thickness and the sequence was ECG-triggered.

Image analysis was performed using Segment software (version v4.0 R12067, Medviso, Sweden) as previously described (13, 14). The regional LV function and wall motion were visually evaluated on cine CMR and quantitatively evaluated using short-axis slices from the base to the apex. The epicardial and endocardial LV borders at end-diastolic and systolic phases were manually delineated and the papillary muscle was excluded from the endocardium. LV end-systolic volume (LVESV), LV end-diastolic volume (LVEDV), stroke volume (SV), LV ejection fraction (LVEF), left ventricular mass (LVM), and LV WT% were evaluated.

### Evaluation of glucose uptake by ^18^F-FDG PET/CT imaging

To evaluate myocardium glucose uptake, ^18^F-FDG PET/CT scan was performed at 1 week after MI and 4, 8 weeks exercise training on a dedicated small animal micro-PET/CT scanner (Inviscan, IRIS PET/CT, France). After anesthetization (2%–3% isoflurane), rats were injected with ^18^F-FDG (6.5 ± 2.1 MBq) through the tail vein. A 10-min static scan was acquired 1 h after injection, followed by a low-dose CT scan for attenuation correction (tube voltage 80 kV; tube current 1 mA). Heart rate, temperature and respiration were continuously monitored. The matrix size was 101 × 101 × 120, and the voxel dimension was 0.85 mm. The field-of-view (FOV) was 80 mm × 95 mm. All PET images were reconstructed using the 3D ordered-subsets implementation of the Monte Carlo algorithm, with 8 subsets and 8 iterations.

The images were analyzed by PMOD software (Version 4.1, Switzerland). In addition, the ^18^F-FDG uptake in the regions of interest was quantitatively determined by measuring the mean standard uptake value (SUV mean) and the maximum standard uptake value (SUV max) of the left ventricle.

The CMR and PET/CT images were visually evaluated by two experienced physicians specializing in cardiac PET imaging. To ensure objectivity and reduce potential bias, the image analysts were blinded to the group assignments during data analysis. Consensus was reached between the two readers on all image interpretations.

### Histology

The hearts (*n* = 4) were harvest and fixed with 10% formalin for 48 h and processed for paraffin histology (4-μm sections stained with Hematoxylin-eosin and Masson's trichrome). Wheat germ agglutinin (WGA) staining was used to measure the cross-sectional area of cardiomyocytes. After deparaffinization and antigen repair with hot EDTA buffer, slides were incubated with WGA conjugated to Alexa Fluor 488 (1 h at 37℃ in a dark incubator). The cross-sectional area of cardiomyocyte was visualized with a Nikon fluorescence upright microscope, and quantified using ImageJ software. For immunohistochemical studies, we investigated the GLUT4 and PFKFB3 which related to glucose metabolism. GLUT4 is an exercise sensitive glucose transporter, which mainly exists in myocardium, skeletal muscle and adipose tissue. It can help glucose molecules enter cells and promote glucose metabolism. PFKFB3 is a key enzyme in the glycolysis pathway that can promote glycolysis and provide energy for the body. The cardiac sections were repaired with hot citrate buffer and blocked with 5% bovine serum albumin (BSA) and then incubated with rabbit polyclonal antibody against GLUT4 (Affinity, AF5386, China) and PFKFB3(Affinity, DF12016, China) overnight at 4℃. Next day, secondary antibody was incubated for DAB chromogenic reaction. Sections were dehydrated and mounted for scanning observation after counter­staining nuclei with hematoxylin. Images were obtained under a Nikon inverted microscope.

### RNA-Seq and quantitative real-time PCR

The cardiac tissues of ischemic border zone and skeletal muscle (*n* = 6) were obtained for RNA-seq analysis. After total RNA extraction, reverse transcription to cDNA, library construction, and sequencing on the NovaSeq 6000 platform (Illumina, U.S.A.), the raw data were obtained. Differential analysis of gene expression was performed using DESeq2 package with log2 fold change ≥1 and *P*-value < 0.05. The cluster profiler package was used for pathway enrichment analysis including gene function enrichment analysis (GO enrichment analysis, KEGG pathway analysis) between sample groups. The quality and quantity of library were confirmed by quantitative real-time PCR (qPCR).1 μg total RNA was used to synthesize cDNA according to the manufacturer's protocol by using 5X Evo M-MLV RT Reaction Mix. The desired cDNA fragments were amplified by qPCR using the selected gene associated primers. The expression levels of each gene were calculated using the $2^{-△△\mathrm{CT}}$ method and fold differences were normalized to the housekeeping gene GAPDH. Primer sequences are listed in Supplementary Tables S1, S2.

### Western blot

To determine protein levels, total protein was extracted from heart tissue (ischemic border zone), following standard protocols as previously described (15). As for primary antibody usage, P-AMPK (AF3423, Affinity, 1:500), GLUT4 (AF5386, Affinity, 1:500), PFKFB3 (DF12016, Affinity, 1:500), GAPDH (200306-7E4, Affinity, 1:500).

### Statistics analysis

All the statistical analysis was performed using SPSS 25.0 and Prism 10.0. RNA-seq data were analyzed using R version 3.6.1. Normal distribution was determined by Shapiro-Wilk test and continuous variables which conform to normal distribution were expressed as mean ± standard deviation (SD). The differences of normal variates were tested by Student's *t*-test (within 2 groups) or one-way analysis of variance (ANOVA, among 3 groups) with *post hoc* comparisons by LSD multiple comparisons test. Data not following a normal distribution were expressed as median and quartile, and analyzed by Mann–Whitney *U* test or Kruskal–Wallis *H* test between groups. A significant difference was considered if the *p* value was less than 0.05.

## Results

### Exercise improved the physiological function in AMI rats

**Figure 1:**
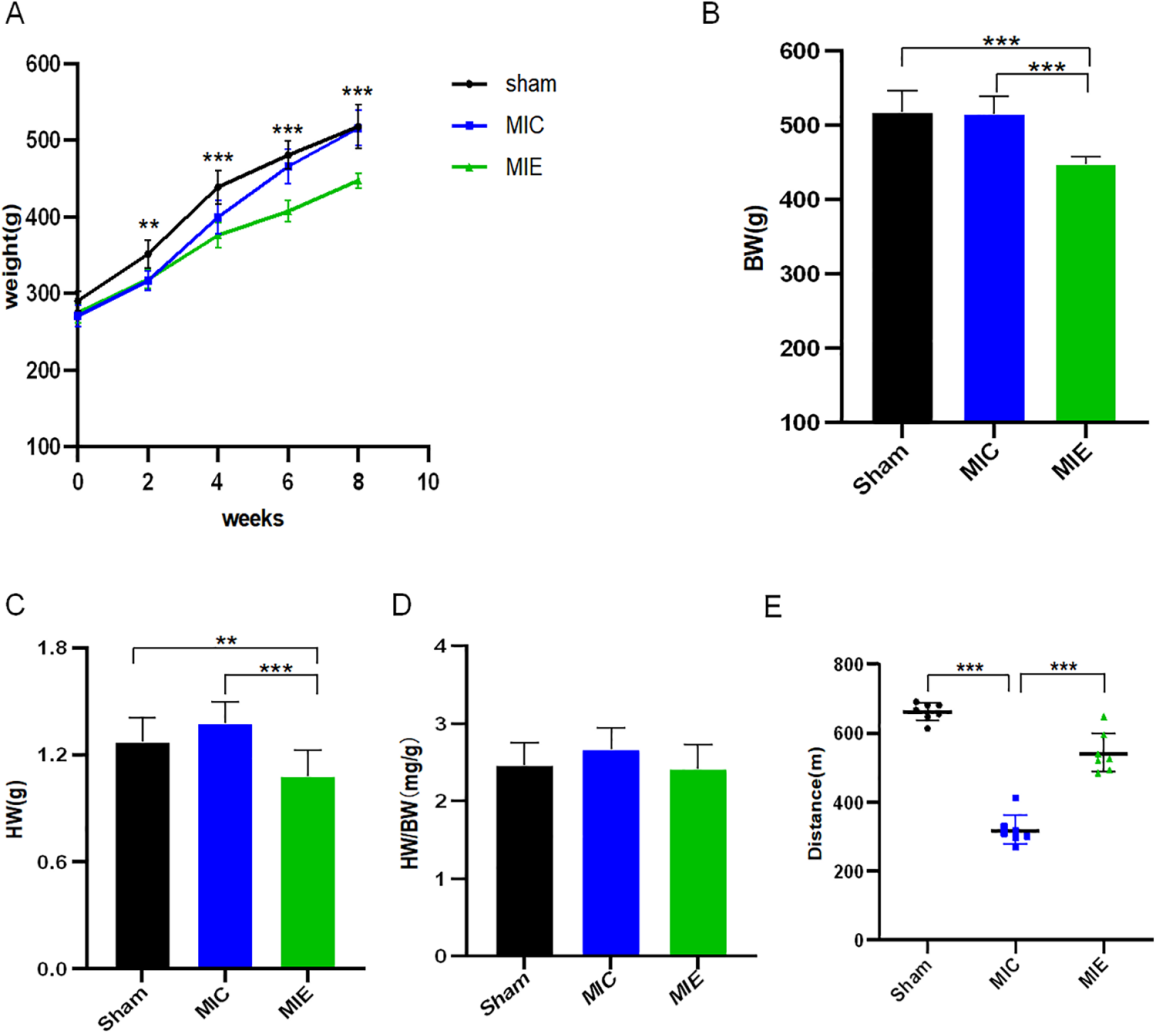
Physiological effects of early exercise training in AMI rats. **(A)** Time course of body weight in the different groups of rats. **(B–D)** Body weight (BW), heart weight (HW), heart weight-to-body weight ratio (HW/BW) were recorded at the 8 weeks of exercise training. **(E)** Quantification of exercise exhaustion test (running distance) of Sham, MIC and MIE rats at the end point of experiment. **P < 0.01, ***P < 0.001. AMI, acute myocardial infarction.

The rats in the exercise group showed a slower increase in body weight compared to the MIC group starting from 4 weeks of exercise, and this trend persisted through 8 weeks. At 4 weeks, BW was significantly lower in the MIE group compared to the MIC group (375.84 ± 16.26 g vs. 399.30 ± 22.07 g, *P* = 0.015). By 8 weeks, this difference became more pronounced (447.35 ± 10.02 g vs. 515.88 ± 23.58 g, *P* < 0.001). Heart weight (HW) was lower in the MIE group compared to both the Sham and MIC groups (*P* < 0.01). However, the heart weight-to-body weight ratio (HW/BW) was similar across the three groups. The treadmill-based exercise exhaustion test revealed that the running distance was significantly longer in the MIE group compared to the MIC group (*P* < 0.001) (Figure 1).

### Exercise improved cardiac function and attenuated myocardial remodeling in AMI rats

CMR assessments confirmed significant left ventricular (LV) dysfunction in rats 7 days post-AMI. Following 4 weeks of exercise training, the exercise training led to significant improvements in cardiac function, as evidenced by an increase in stroke volume (SV) and cardiac output (CO). This was accompanied by a notable enhancement in left ventricular ejection fraction (LVEF), reflecting improved overall heart function. Additionally, the exercise training resulted in a significant reduction in left ventricular end-systolic volume (LVESV), indicating a more efficient heart contraction, while left ventricular end-diastolic volume (LVEDV) showed a slight increase, suggesting better filling capacity. These positive changes were maintained over 8 weeks exercise training, indicating sustained improvements in cardiac performance (Table 1).

**Table 1:** MRI parameters measured at 4 and 8 weeks of exercise training between groups.

	4w			8w		
	Sham (7)	MIC (7)	MIE (6)	Sham (7)	MIC (7)	MIE (6)
LVEDV(μl)	427.94 ± 71.77	373.11 ± 61.81	397.34 ± 81.40	423.71 ± 45.29	379.83 ± 86.90	485.37 ± 39.27**
LVESV(μl)	153.84 ± 27.05	262.77 ± 63.52*	210.96 ± 39.40*	153.06 ± 25.25	212.31 ± 43.69*	257.02 ± 28.85*^,^**
SV(μl)	274.09 ± 51.98	110.34 ± 25.95*	186.37 ± 45.39*^,^**	270.64 ± 40.56	167.52 ± 46.89*	259.43 ± 27.53**
CO(μl/min)	76.74 ± 14.55	30.89 ± 7.26*	52.18 ± 12.71*^,^**	75.78 ± 11.35	46.90 ± 13.13*	53.69 ± 31.84
LVEF (%)	63.89 ± 3.74	30.10 ± 7.62*	46.67 ± 2.94*^,^**	63.77 ± 5.34	43.77 ± 3.61*	53.51 ± 2.82*^,^**
LVM(g)	0.71 ± 0.14	0.85 ± 0.09	1.04 ± 0.17*^,^**	0.63 ± 0.13	0.91 ± 0.13*	0.86 ± 0.85*
WT (%)						
Apical anterior	57.03 ± 23.41	1.93 ± 25.39*	19.59 ± 24.81*	55.28 ± 21.87	5.06 ± 16.18*	30.16 ± 30.65
Apical septal	54.19 ± 11.60	11.21 ± 17.99*	33.16 ± 15.58*^,^**	53.68 ± 10.67	12.25 ± 9.60*	35.95 ± 23.96**
Apical inferior	70.59 ± 26.19	16.02 ± 6.93*	37.63 ± 14.39*	78.48 ± 31.74	22.02 ± 15.88*	36.91 ± 7.50*
Apical lateral	46.34 ± 27.82	7.46 ± 18.03*	20.29 ± 21.95	50.49 ± 27.66	11.15 ± 15.23*	26.69 ± 17.77
Apex	28.44 ± 33.34	2.03 ± 19.71*	15.36 ± 18.52	29.60 ± 30.59	15.68 ± 17.57	34.42 ± 19.61

Comparison between MIC and Sham. *P < 0.05. Comparison between MIE and MIC: **P < 0.05.

**Figure 2:**
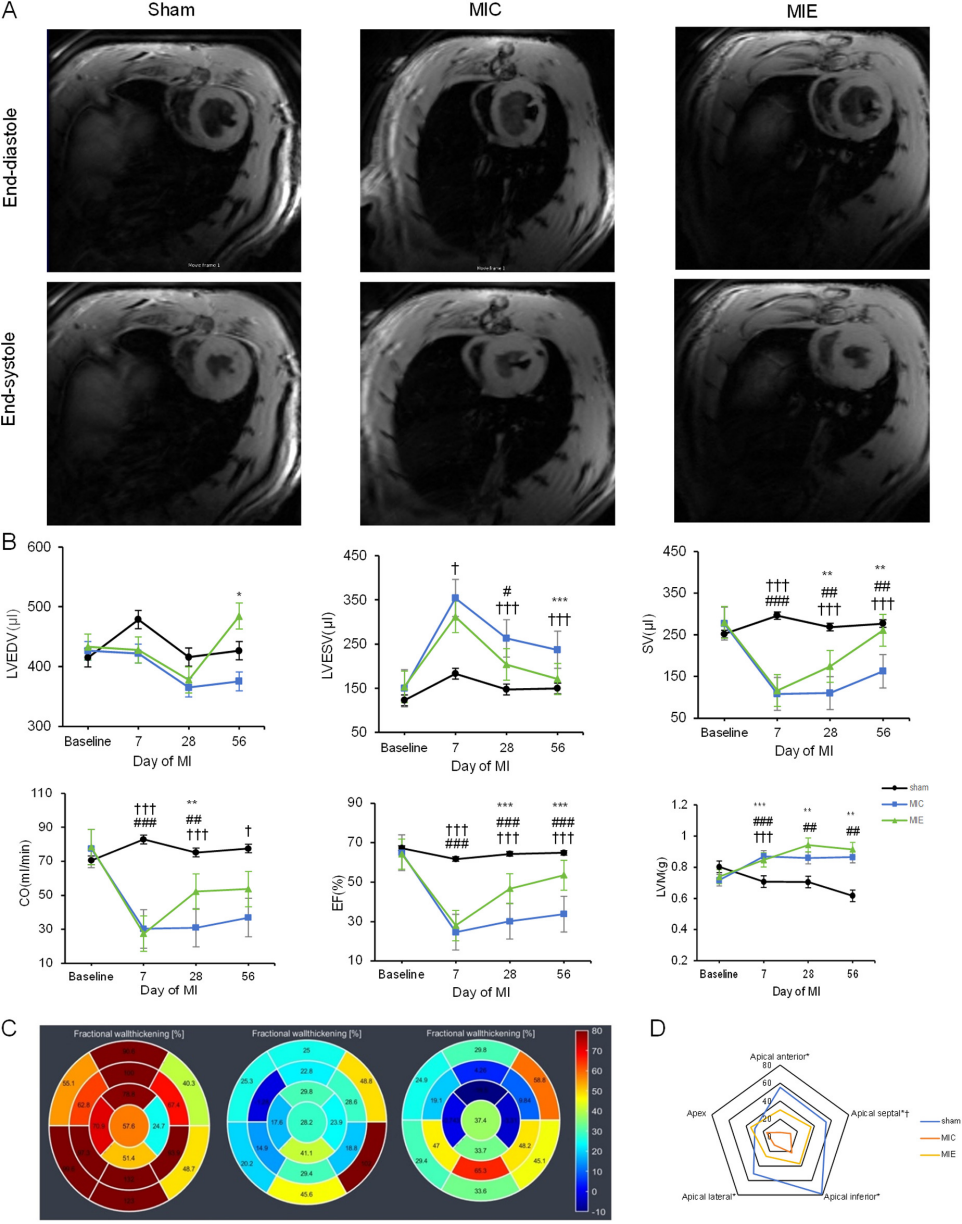
Effects of exercise training on cardiac function measured by CMR. **(A)** Representative short-axis slices from cine CMR at end-diastole (top) and end-systole (bottom) of three groups after 8 weeks of exercise. **(B)** Time course of LV parameters in the three groups. Rats in the MIC and MIE groups underwent significant drops in LVEDV, SV, CO and LVEF compared to the sham group. LVEDV and LVEF increased obviously in the MIE group compared to the MIC group with the continued exercise training. **(C)** Representative 17-segment CMR data, arranged from left to right, show the Sham, MIC, and MIE groups at 8 weeks of exercise. Color and figure are representative the WT%. **(D)** WT% between end-diastole and end-systole demonstrated for each group. Blue lines represent the Sham group, yellow lines represent the MIE group and red lines represent the MIC group. Comparison between MIC and Sham: *P < 0.05. Comparison between MIE and MIC: ^†^*P* < 0.05. LVEDV, left ventricular end-diastolic volume; SV, stroke volume; CO, cardiac output; LVEF, left ventricular eject fraction; WT%, wall thickening percentage.

CMR data were analyzed using the 17-segment polar map model to evaluate WT%. A representative polar map revealed reduced LV WT% in the apical anterior, apical septal, apical inferior, and apical lateral segments of the MIE group. By 8 weeks, these segments showed significant recovery in the MIE group (Figure 2).

**Figure 3:**
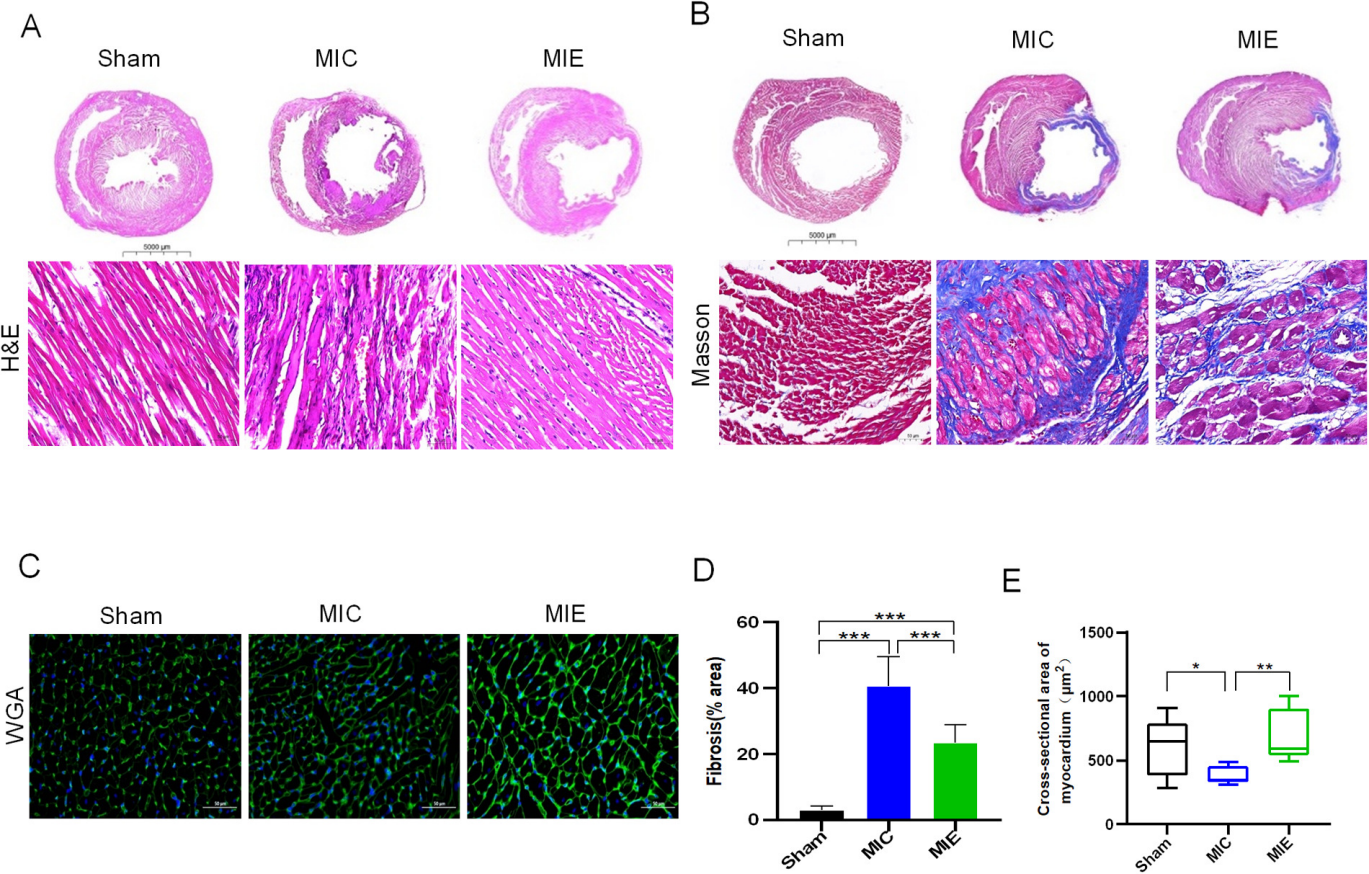
Heart morphology and pathology of rats after 8 weeks of exercise. Representative images of hematoxylin-eosin (H&E) **(A)**, Masson's trichrome **(B)** and WGA **(C)** in transversal sections of left ventricle of rats of the three groups. Scale bars, 5,000 μm (scale bar of the gross pathology slides of H&E, Masson's trichrome) and 50 μm (scale bar of the local pathology and WGA).

Histopathological analysis highlighted reductions in inflammatory cell infiltration and fibrosis in the MIE group compared to the MIC group (23.42 ± 5.4% vs. 40.63 ± 8.9%, *P* < 0.05). Wheat germ agglutinin (WGA) staining indicated increased cross-sectional myocardial area in the MIE group (817.15 ± 36.54 μm² vs. 379.28 ± 67.99 μm², *P* = 0.002) (Figure 3).

### Exercise enhanced myocardial glucose metabolism in AMI rats

**Figure 4:**
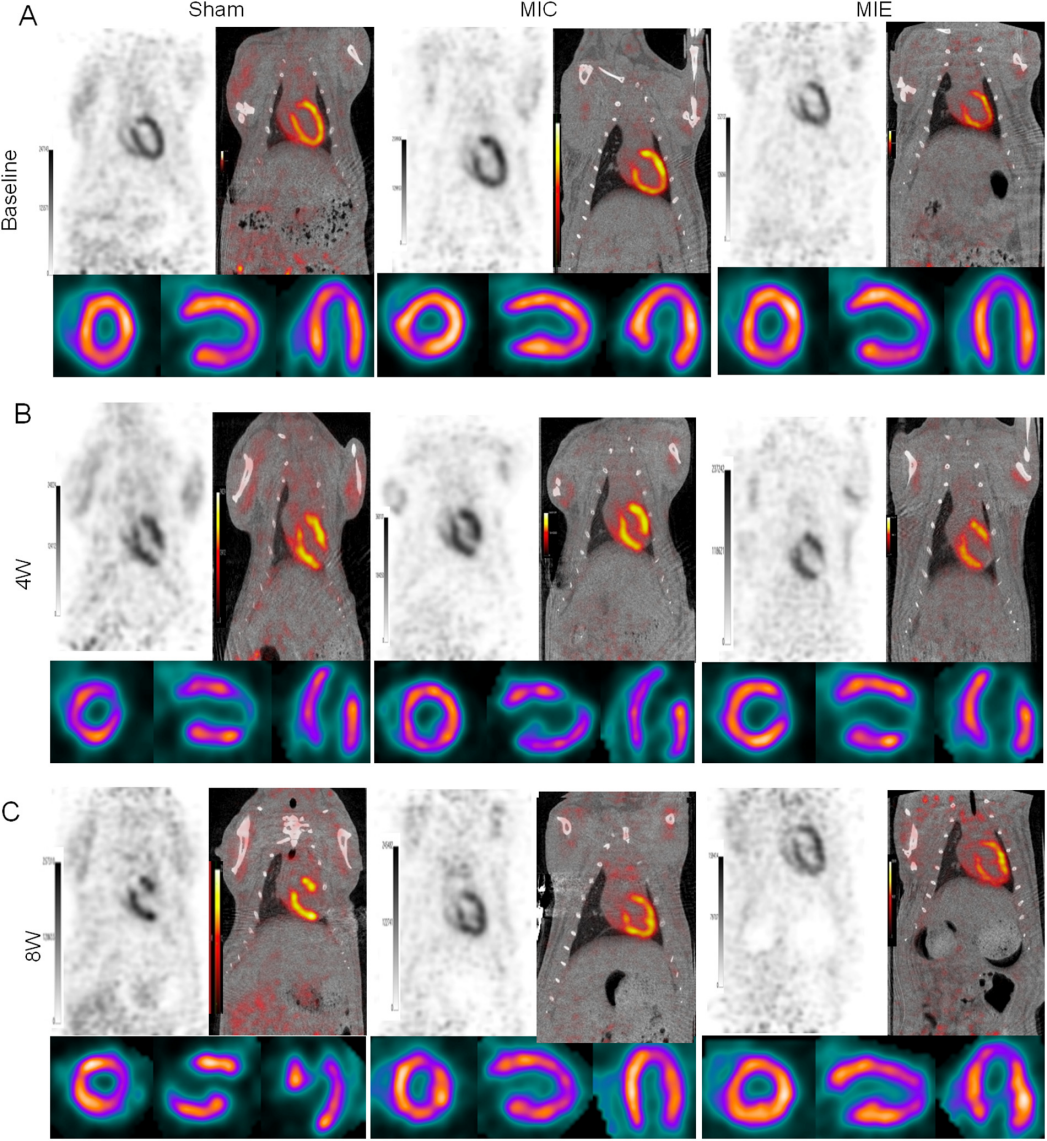
Changes of glucose uptake after AMI and effects of exercise. Myocardium glucose metabolism were assessed by ^18^F-FDG uptake using micro-PET at baseline (1 week after AMI), and 4, 8 weeks exercise training. Representative coronal, short axis, horizontal long-axis and vertical long-axis of ^18^F-FDG uptake in the three groups.

**Figure 5:**
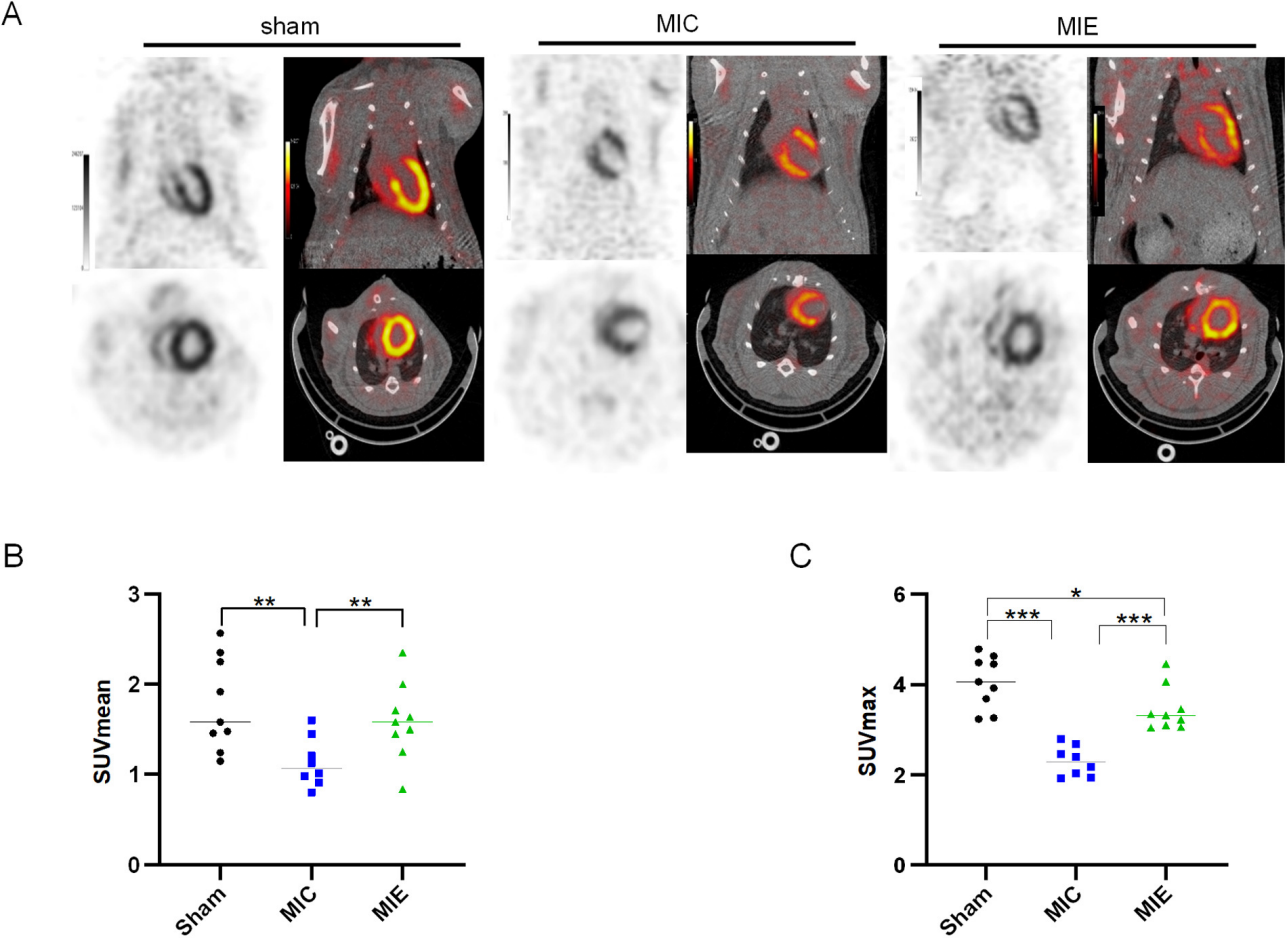
Quantitative analysis of ^18^F-FDG PET imaging. **(A)** Representative PET/CT images of the three groups of rats in the coronal and transverse planes after 8 weeks exercise training. **(B,C)** Quantitative analysis of SUV mean and SUV max achieved from ^18^F-FDG PET/CT scans in the three groups. *P < 0.05, **P < 0.01, *** P < 0.001.

**Figure 6:**
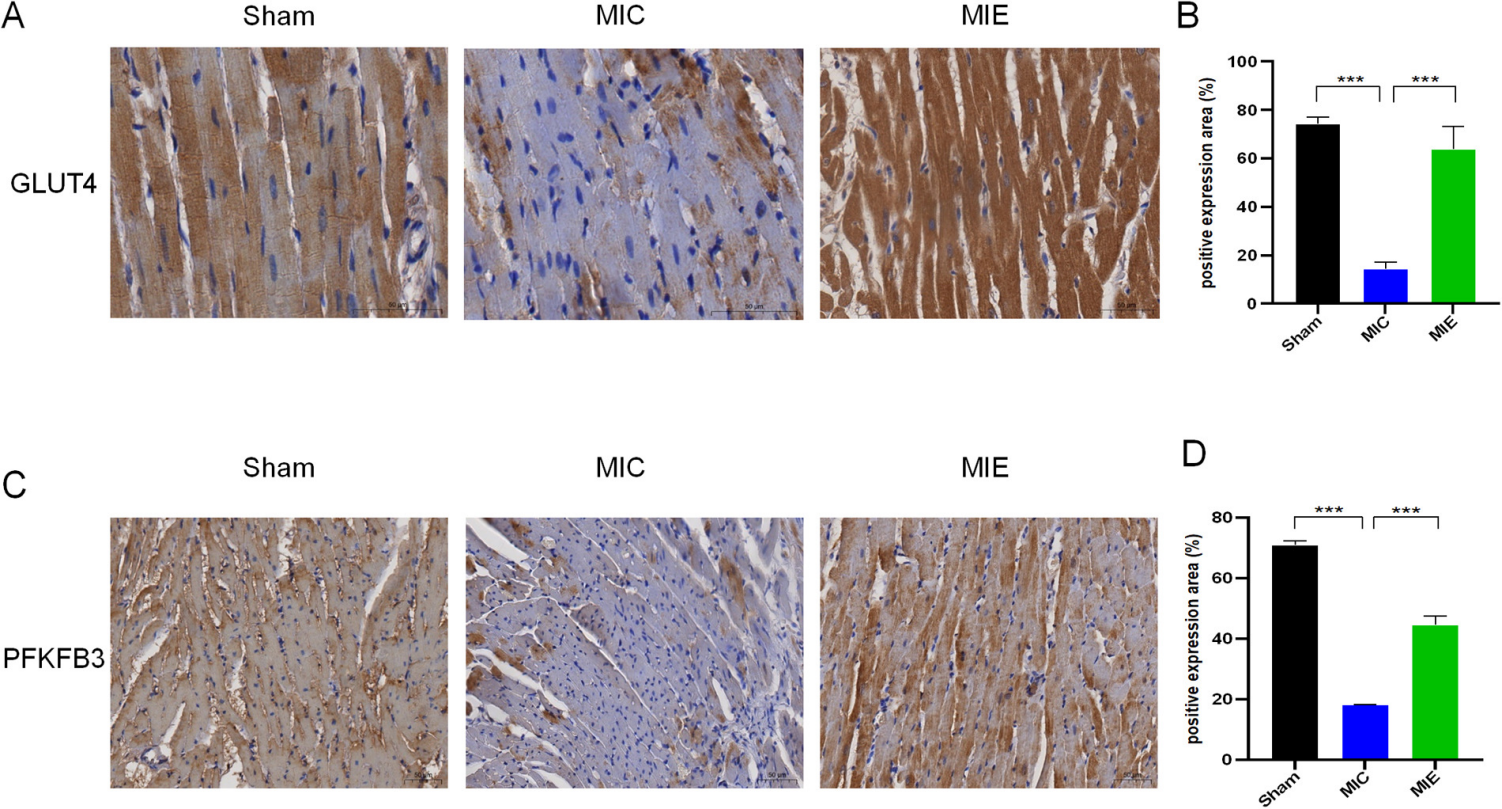
Effects of exercise on the expressions of GLUT4 and PFKFB3 in myocardial tissues of rats after 8 weeks of exercise training. **(A,C)** Representative micrographs of heart sections from the ischemic border zones of the three groups. **(B,D)** Protein expressional levels of GLUT4 and PFKFB3 in the Sham, MIC and MIE groups. *n* = 4 rats per group. ***P < 0.001.

^18^F-FDG PET/CT imaging demonstrated that SUV max significantly increased in the ischemic border myocardium of the MIE group after 4 weeks of exercise training (Table 2). Both SUV mean and SUV max showed further enhancement after 8 weeks. Representative PET/CT images at baseline (7 days post-AMI), 4 weeks, and 8 weeks of exercise training are shown in Figure 4. By 8 weeks, the MIE group exhibited significantly higher ^18^F-FDG uptake in the ischemic border myocardium compared to the MIC group (*P* = 0.037 for SUVmean, *P* < 0.001 for SUVmax) (Figure 5). Consequently, exercise training significantly upregulated GLUT4 and PFKFB3 expression in the MIE group compared to the MIC group (MIE vs. MIC, all *P* < 0.05) (Figure 6).

**Table 2:** Longitudinal evolution of [^18^F] F-FDG PET/CT uptake in the ischemic border myocardium.

Timepoint	Group	SUV mean	SUV max
MI+7d	Sham	2.10 ± 0.59	5.00 ± 0.60
	MI	1.25 ± 0.31*	2.68 ± 0.51*
MI+4w	Sham	2.47 ± 0.99	5.25 ± 0.78
	MIC	1.33 ± 0.24*	2.64 ± 0.52*
	MIE	2.07 ± 0.43	3.38 ± 0.23*^,^**
MI+8w	Sham	1.77 ± 0.51	4.05 ± 0.57
	MIC	1.13 ± 0.27*	2.30 ± 0.33*
	MIE	1.59 ± 0.43**	3.44 ± 0.48*^,^**

Comparison between MIC and Sham: *P < 0.05. Comparison between MIE and MIC: **P < 0.05.

### Transcriptome changes mediated by exercise post-AMI

**Figure 7:**
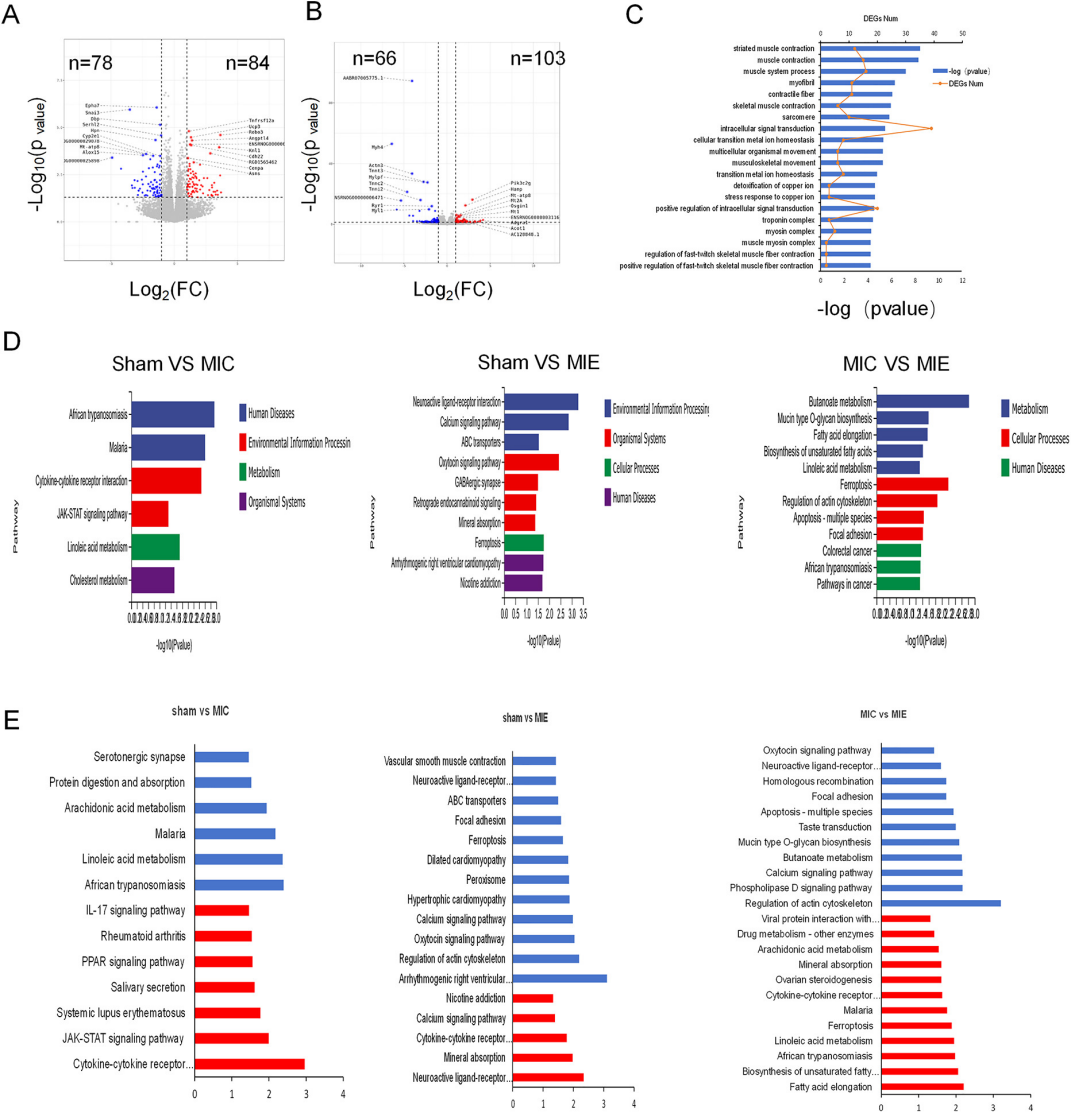
Results from RNA-Seq of myocardial tissue. **(A,B)** Volcano plots showing different expression genes (DEGs) between Sham and MIC, MIC and MIE respectively. **(C)** Twenty significantly enriched categories were identified through functional enrichment analysis of gene ontology (GO). Red line: Number of DEGs. Bars: Significance level. **(D)** The most highly significant pathways differentially regulated in metabolic pathways and cellular processes among the three groups. **(E)** KEGG enrichment analysis of metabolism pathways for genes showing upregulation (red) and downregulation (blue) in Sham, MIC and MIE rats. For volcano plots, grey represents nonsignificant, red indicates upregulated genes with *P* < 0.05, blue indicates downregulated genes with *P* < 0.05.

RNA-seq analysis revealed significant differential gene expression in the MIE group after exercise training. Functional enrichment analysis identified 20 gene ontology (GO) categories, with the most significant enrichment in pathways related to muscle contraction, calcium release channel activity, and intracellular signal transduction. Kyoto Encyclopedia of Genes and Genomes (KEGG) pathway analysis showed enrichment in pathways such as butanoate metabolism, mucin-type O-glycan biosynthesis, and fatty acid reinforcement (Figure 7).

Comparative transcriptomic analysis of skeletal muscle identified GO categories significantly enriched for inflammation, stress response, fatty acid metabolism, cytokine production, and extracellular matrix production in the MIE group. KEGG analysis highlighted upregulated pathways including AMPK signaling, cAMP signaling, cytokine receptor activity, and Ca^2+^ regulation. Conversely, pathways related to PPAR signaling, IL-17-mediated inflammation, and lipid digestion were downregulated in the MIE group compared to the MIC group (Supplementary Figure S1).

**Figure 8:**
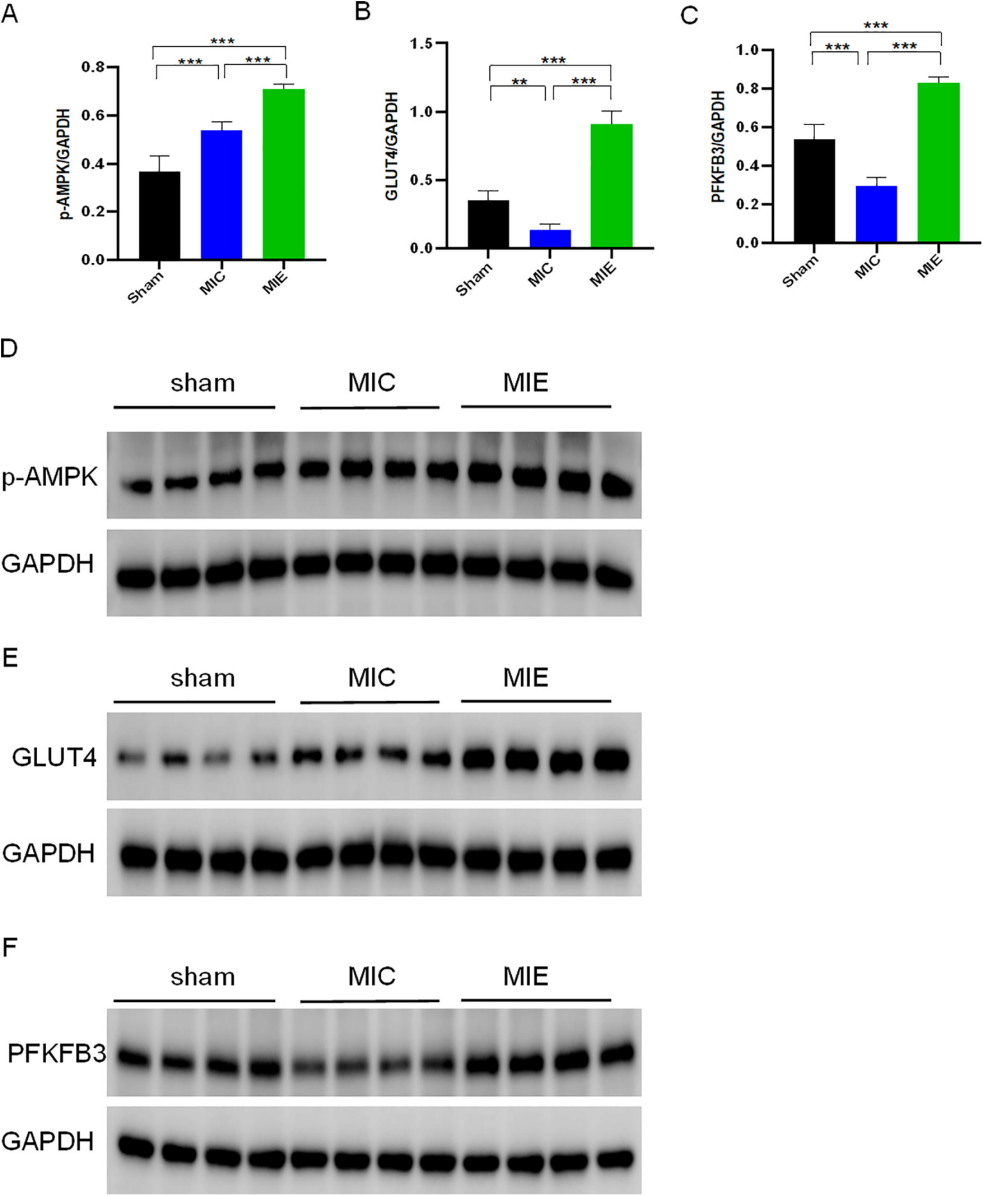
Effects of exercise training on metabolic pathways in myocardial tissue of rats after AMI. Protein expression levels of p-AMPK **(A)**, GLUT4 **(B)**, and PFKFB3 **(C)** in myocardial tissue of rats from three groups after 8 weeks of exercise training, as determined by Western blotting. Protein levels were normalized to glyceraldehyde 3-phosphate dehydrogenase (GAPDH). Representative Western blot images for p-AMPK **(D)**, GLUT4 **(E)**, and PFKFB3 **(F)** are shown for each group. *n* = 4/group. **P < 0.01, **P < 0.001.

Real-time qPCR analysis validated RNA-seq findings, showing increased mRNA expression of key metabolic genes in the MIE group (Supplementary Figure S2). Western blot analysis further confirmed increased protein expression of p-AMPK, GLUT4, and PFKFB3 in myocardial tissue of the MIE group compared to the MIC and Sham groups (Figure 8).

## Discussion

Physical activity is associated with improved survival rates following cardiovascular events compared to a sedentary lifestyle (16, 17). Although previous studies have discussed the benefits of exercise for heart failure patients (18, 19), the impact of exercise training after acute myocardial infarction (AMI) remains a topic of debate among clinicians. Some studies suggest that early exercise may be detrimental, reducing overall survival rates (20). Conversely, a 5-year follow-up study demonstrated that early initiation of exercise therapy in post-MI patients significantly reduced cardiovascular hospitalization rates and mortality (8). Therefore, our study aimed to evaluate the effects of early exercise training on cardiac function following AMI.

Unlike previous studies that predominantly relied on echocardiographic evaluation, we utilized a 7T magnetic resonance imaging system with a dedicated cardiac coil. This approach enabled precise assessment of cardiac structure and ventricular volumes.

Based on CMR findings, 4 weeks of exercise training lead to cardiac chamber dilation and an decrease in LVESV. These structural changes contributed to significant improvements in stroke volume, cardiac output and oxygen delivery. By 8 weeks, increased LVEDV in the exercise group, compared to controls, suggested enhanced venous return and improved cardiac preload. These observations align with findings from Havard et al. regarding enhanced systolic and diastolic function in athletes (21).

Previous reports have described that exercise downregulated MMP9, leading to a reduction in fibrosis and matrix degradation, thereby regulating cardiac remodeling (22). In the present study, using a 17-segment CMR model, we mapped fractional wall thickening (WT%) and observed recovery in segments surrounding the injured myocardium in the exercise group after 8 weeks. This recovery was corroborated by histopathological analysis, which revealed reduced fibrosis in the myocardial tissue, highlighting the advantages of CMR over traditional echocardiography in detecting both segmental and global LV function changes.

To investigate the metabolic adaptations associated with exercise, we assessed glucose uptake using ^18^F-FDG PET/CT. Our findings showed significant increases in glucose uptake in the ischemic border zone of the exercise group compared to sedentary controls at 4 weeks, which persisted through 8 weeks. This increased glucose utilization is likely attributable to hypoxic conditions in the ischemic myocardium, where limited oxygen availability reduces lipid metabolism and shifts the primary substrate to glucose for ATP production.

It is important to note that rodents and humans differ in their predominant cardiac metabolic substrates. These differences highlight the need for caution when extrapolating findings from animal experiments to human clinical settings. Investigating how exercise training affects glucose metabolism in cardiomyocytes under ischemic conditions in humans will be the focus of our future research. Nonetheless, it is worth emphasizing that the treadmill protocol used in our study corresponds to moderate-intensity aerobic exercise, which lays a valuable foundation for potential clinical application in myocardial infarction patients.

The present study identified an increased expression of the glucose transporter 4 (GLUT4) in cardiomyocyte. This phenomenon might be explained by the fact that exercise could activate AMPK phosphorylation (23, 24) which promotes GLUT4 translocation, enhancing glucose metabolism and improving cardiac function in AMI rats. Additionally, exercise increased the expression of 6-phosphofructo-2-kinase/fructose-2,6-bisphosphatase 3 (PFKFB3), a key glycolytic enzyme. RNA-seq data confirmed increased PFKFB3 activity following AMPK activation, and these findings were validated by qPCR and western blot analyses. While previous studies have reported reduced PFKFB3-dependent glycolysis in aging microglia, our results suggest that hypoxic conditions in the myocardium favor glycolysis to meet energy demands (25). This differs from our findings, possibly due to the differences of cellular environment. Under hypoxic conditions, cells tend to favor metabolic pathways with lower oxygen consumption, utilizing glycolysis to provide more ATP for the body. Although it is reported that the fatty acid oxidation of healthy subjects increases after exercise (26). Meanwhile, we observed a decrease in mRNA expression of carnitine palmitoyltransferases 1 and 2 (CPT1 and CPT2), critical enzymes for β-oxidation, following exercise training. This reduction may reflect a shift away from fatty acid metabolism post-MI.

In addition, we found that in skeletal muscle tissue, exercise-trained rats exhibited significant upregulation of pathways related to AMPK signaling, cAMP signaling, cytokine-receptor interactions, Ca^2+^ regulation, and glucagon signaling. Conversely, pathways related to PPAR signaling, inflammatory factors (such as IL-17), and lipid digestion and absorption were markedly downregulated. These findings may provide valuable insights for future research on the application of exercise training in age-related muscle atrophy.

To the best of our knowledge, this is the first experimental to investigate exercise-induced transcriptomic changes in an AMI model. RNA-seq revealed that exercise downregulated apoptosis-related pathways, consistent with findings from studies on aging rats (27). The RNA-seq results revealed notable pathway changes following exercise training. Specifically, there was an upregulation in pathways such as AMPK signaling, cAMP signaling, cytokine receptors, Ca^2+^ regulation, and glucagon signaling. In contrast, pathways related to PPAR signaling, inflammatory factors (e.g., IL-17), and lipid digestion and absorption were downregulated.

### Limitations

Several limitations should be noted. First, this study exclusively used male rats, leaving potential sex differences in myocardial infarction and exercise responses unaddressed. Second, while RNA-seq provided valuable insights into transcriptomic changes, it did not account for regulatory changes at the protein or post-translational levels. Future studies incorporating metabolomics will help elucidate the broader metabolic effects of exercise. Lastly, the exercise regimen used in this study may not directly translate to human scenarios, necessitating further research for clinical application.

## Conclusions

Our study demonstrated that early exercise training after AMI is both effective and safe, as evidenced by improvements in cardiac function, myocardial remodeling, and glucose metabolism. Advanced imaging modalities such as CMR and PET provided precise evaluations of these changes. Our findings suggested that early exercise-induced upregulation of GLUT4 and PFKFB3 may represent key molecular targets for enhancing cardiac recovery following AMI. Future research will evaluate the long-term impact of exercise training. In addition, we will focus on exploring these mechanisms further and identifying therapeutic targets that mimic the effects of exercise, potentially leading to novel treatments for elderly AMI patients.

## Data Availability

The datasets presented in this study can be found in online repositories. The names of the repository/repositories and accession number(s) can be found in the article/Supplementary Material.
