# Supplementary material for: Effect of exercise training on cardiac function and glucose metabolism in the ischemic border zone: insights from multi-modal imaging techniques
Source: Front Cardiovasc Med. 2025 May 30;12:1583206. doi: 10.3389/fcvm.2025.1583206 (PMC12162551; doi:10.3389/fcvm.2025.1583206)
Supplement: Supplementary file 1 [file Datasheet1.docx]

**Effect of Exercise Training on Cardiac Function and Glucose Metabolism in the Ischemic Border Zone: Insights from Multi-Modal Imaging Techniques**


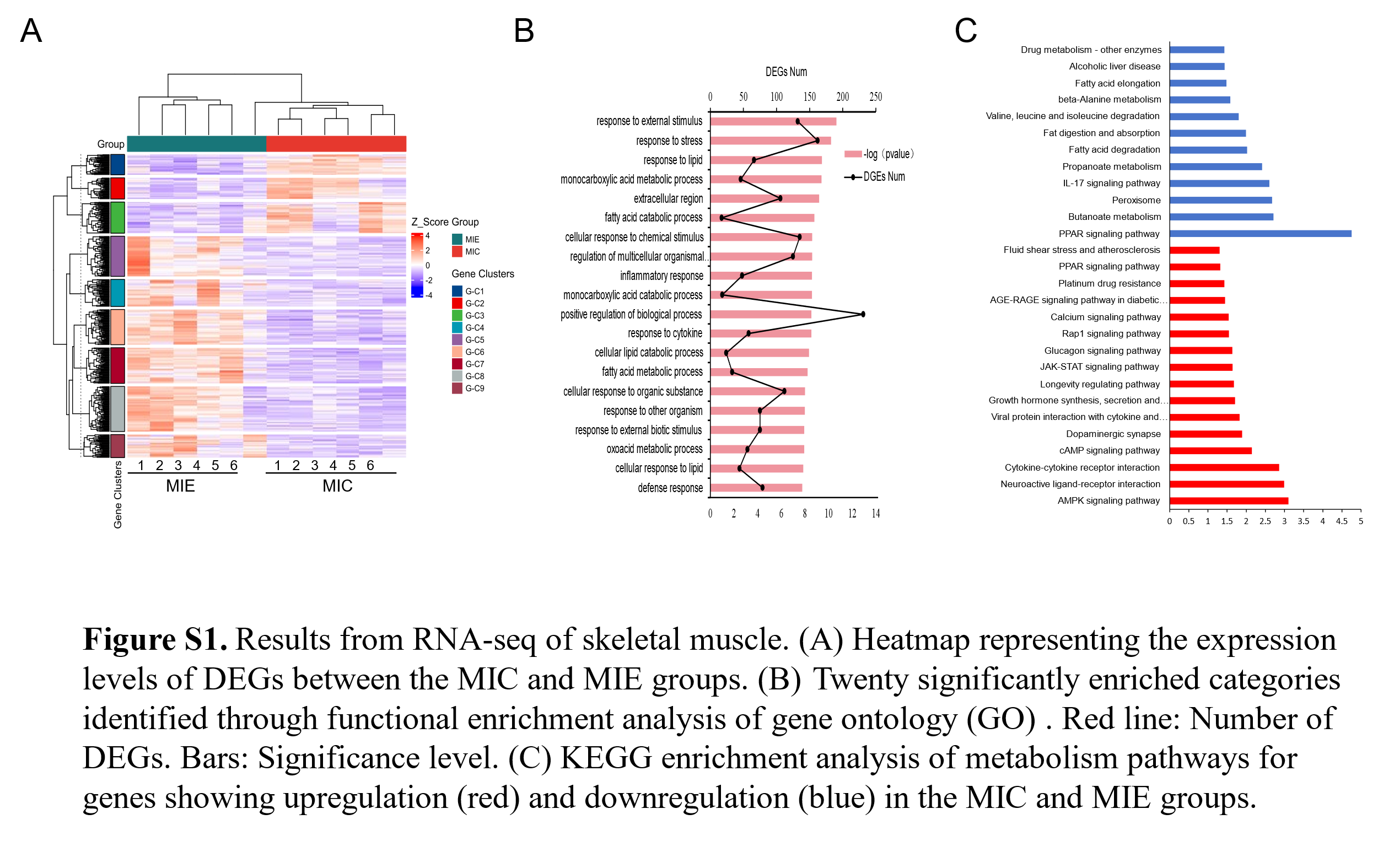


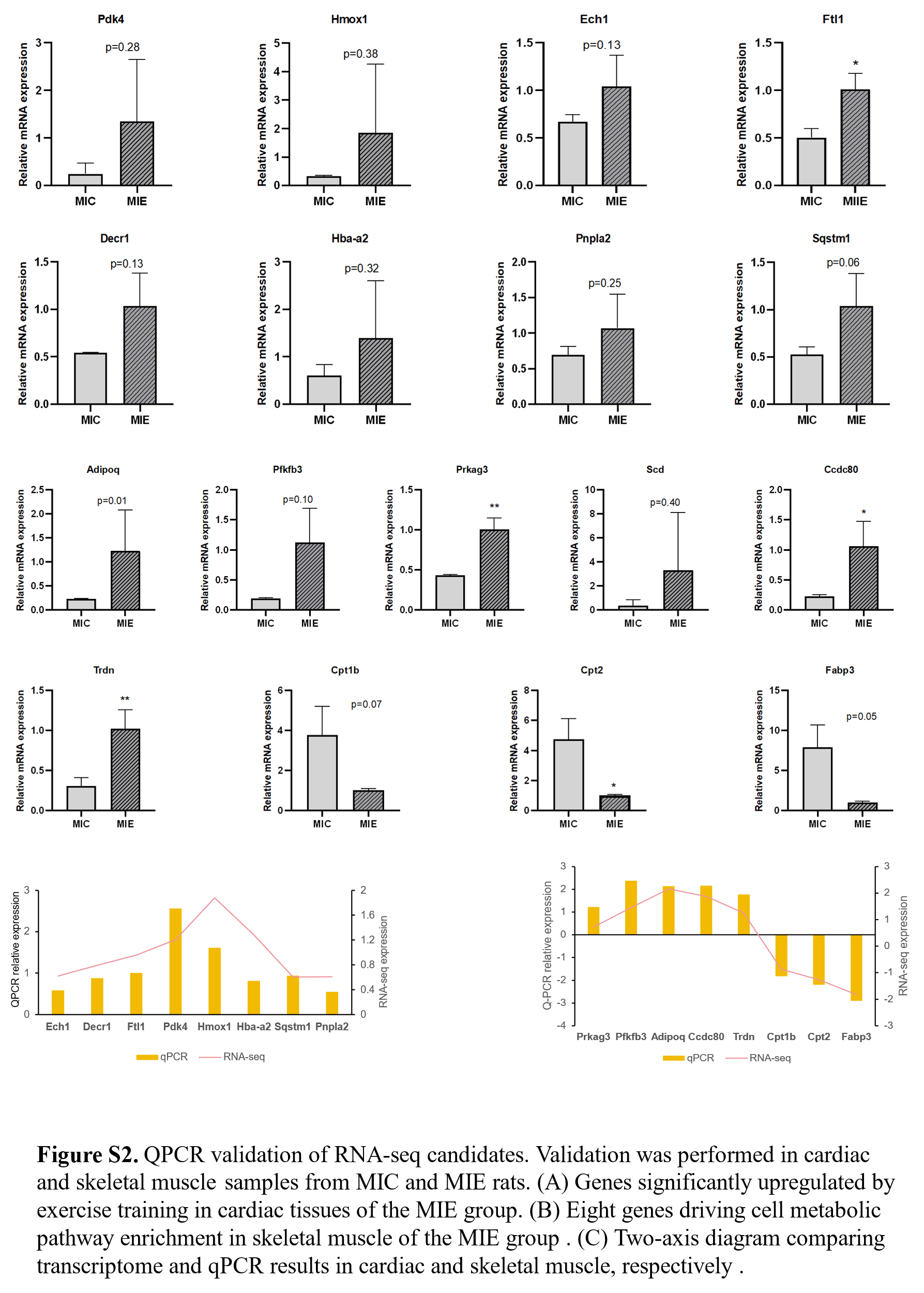


Table S1: Primer sequences used for qPCR in cardiac tissues

| Target name | Forward sequence | Reverse sequence |
| --- | --- | --- |
| Pdk4 | GAGCATCAAGAAAACCGCCC | AGGGGCATTCCGTGAATTGT |
| Hmox1 | GAGCGAAACAAGCAGAACCC | CACCTCGTGGAGACGCTTTA |
| Ech1 | CCCGAGACCATTCTGTGGAC | GCTGCCTGGACTGACTTGAT |
| Mt-atp8 | ATGCCACAACTAGACACATCC | GGCTATAGTTTTGGGGGAGGG |
| Ftl1 | CACCTACCTCTCTCTGGGCT | CAATTCGCGGAAGAAGTGGC |
| Decr1 | AGGCGTGGAAGCCATGAATAA | CCACAGGGGATTCTCTCGAT |
| Hba-a2 | GGCCCTACAGAGGATGTTCG | GCATCAGCAACCTTCTTGCC |
| Pnpla2 | TCCTCGGGGTCTACCACATT | CCGTAGATGTGAGTGGCGTT |
| Sqstm1 | ACGACTGGACGCATTTGTCT | TGGTGGGAGATGTGGGTACA |
| GAPDH | TGACATCAAGAAGGTGGTGAAGC | GGAAGAATGGGAGTTGCTGTTG |

Table S2: Primer sequences used for qPCR in skeletal muscle tissue

| Target name | Forward sequence | Reverse sequence |
| --- | --- | --- |
| Adipoq | GCCGTTCTCTTCACCTACGA | CCCCTTCCCCATACACTTGG |
| Pfkfb3 | TTTGGAACTGACCCAGAGCC | GAGCCCCACCATCACAATCA |
| Foxo1 | GTTTAACCAGTCCAACTCGACC | CGAAGTCCTCGCTCTCTTCTA |
| Prkaa2 | GAAGATCGGACACTACGTGCT | AACTGCCACTTTATGGCCTG |
| Prkag3 | CTGGAGCACACACTGCCTG | ACTCCCCGTATAGCACAGGT |
| Scd | TTCGTCAGCACCTTCTTGAGAT | GTAGTTGTGGAAGCCCTCGC |
| Cpt1b | CATGTATCGCCGCAAACTGG | CCTGGGATGCGTGTAGTGTT |
| Cpt2 | GACCAAAGAAGCAGCGATGG | GTTCAGAGTGCTGGTGGACA |
| Fabp3 | TCAAGTCGGTCGTGACACTG | CCATGGGTGAGAGTCAGGATG |
| Col3a1 | GCCTACATGGATCAGGCCAA | CATGGCCTTGCGTGTTTGAT |
| Ccdc80 | GGGCAAGTTTAGCATGGTGC | TCTGACTGGGTAGGGGTAGC |
| Trdn | GAGAAGTCAGAGCCTCAAGTT | CTGTTTGCGTGTTTGGCCTT |
| GAPDH | TGACATCAAGAAGGTGGTGAAGC | GGAAGAATGGGAGTTGCTGTTG |
